# Providing Several Skills to Treat Complex Infectious Stones of Solitary Kidney in a Patient Failed to Undergo Percutaneous Nephrolithotomy: A Case Report

**DOI:** 10.3389/fsurg.2021.743813

**Published:** 2021-09-29

**Authors:** Dechao Feng, Wuran Wei

**Affiliations:** Department of Urology, Institute of Urology, West China Hospital, Sichuan University, Chengdu, China

**Keywords:** staghorn stones, infectious stones, percutaneous nephrolithotomy, flexible ureteroscopy, case report

## Abstract

Conservative treatment is closely associated with renal deterioration for patients with renal staghorn stones. It is well-recognized that percutaneous nephrolithotomy (PCNL) is recommended as the first-line treatment of renal stones larger than 2 cm due to its higher stone clearance and cost-effectiveness when compared with other treatment alternatives, such as shockwave lithotripsy and flexible ureteroscopy (FURS). Besides, our findings indicated that miniaturized PCNL could be served as an alternative to PCNL with a higher stone-free rate and a lower hemorrhage risk. Despite the higher cost-effectiveness of PCNL, the management of staghorn stones are still controversial in some special situations, such as a solitary kidney. Herein, we present a case with complex infectious stones of a right-sided solitary kidney, complaining of persistent pain in the right waist. The rarity of this case is that it is difficult to encounter these cotton-like staghorn stones which are clinically resistant to holmium laser lithotripsy, and the particularity is that the patient with solitary kidney failed to undergo PCNL. We found that the combination of intermittently high-frequency oscillation and flexible ureteroscopy forceps might contribute to treat the complex infectious stones in a patient with solitary kidney. Our surgical experience might be beneficial to such patients undergoing flexible ureteroscopy in clinical practice.

## Introduction

Conservative treatment is closely associated with renal deterioration for patients with staghorn calculus (1). It is well-recognized that percutaneous nephrolithotomy (PCNL) is recommended as the first-line treatment of renal stones larger than 2 cm due to its higher stone clearance and cost-effectiveness when compared with other treatment alternatives, such as shockwave lithotripsy and flexible ureteroscopy (FURS) (2, 3). Besides, our findings indicated that the miniaturized PCNL could be served as an alternative to PCNL with a higher stone-free rate and a lower hemorrhage risk (4). Despite the higher cost-effectiveness of PCNL, the management of staghorn stones are still controversial in some special situations, such as solitary kidney.

Herein, we present a case with complex infectious stones of a right-sided solitary kidney. The particularity of this case is that the patient has failed to undergo PCNL and the stones are resistant to holmium laser lithotripsy. Our surgical experience might be beneficial to such patients undergoing FURS. We present the following article in accordance with the CARE reporting checklist (5).

## Case Presentation

**Figure 1:**
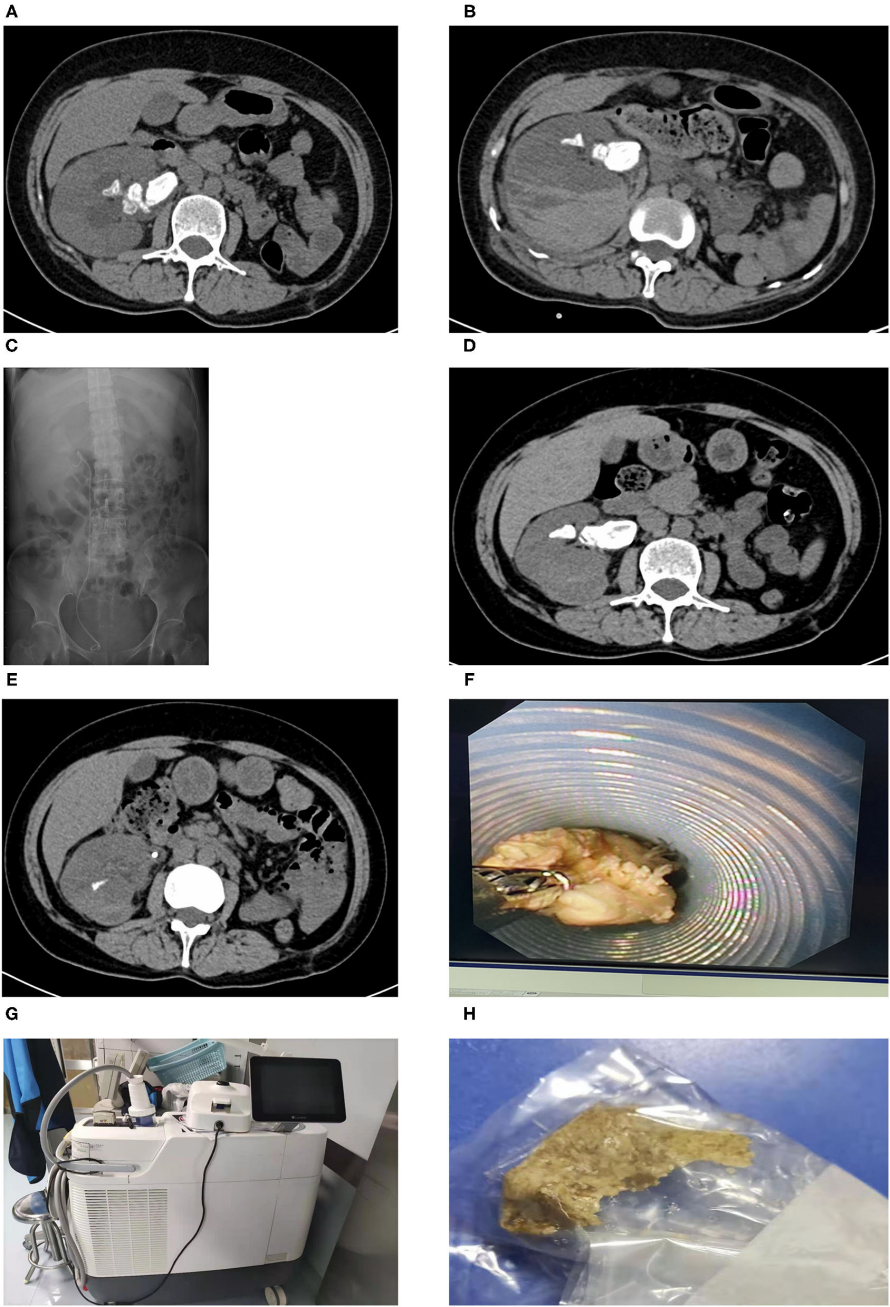
The CT images throughout the period: **(A)** The abdominal CT at admission showing increased right kidney and staghorn stones among which the larger stone was about 3.0 × 1.8 cm; **(B)** the abdominal CT after PCNL of right renal stones, which was terminated due to severe bleeding during channel establishment; **(C)** the abdominal KUB film after right ureteral stent placement; **(D)** the abdominal CT at readmission; **(E)** the postoperative abdominal CT of the third flexible ureteroscopy; **(F)** the image of flexible ureteroscopy forceps grabbing floppy stone; **(G)** Lumenis Pulse 120H; **(H)** infectious stones after the third procedure. PCNL, percutaneous nephrolithotomy.

**Figure 2:**
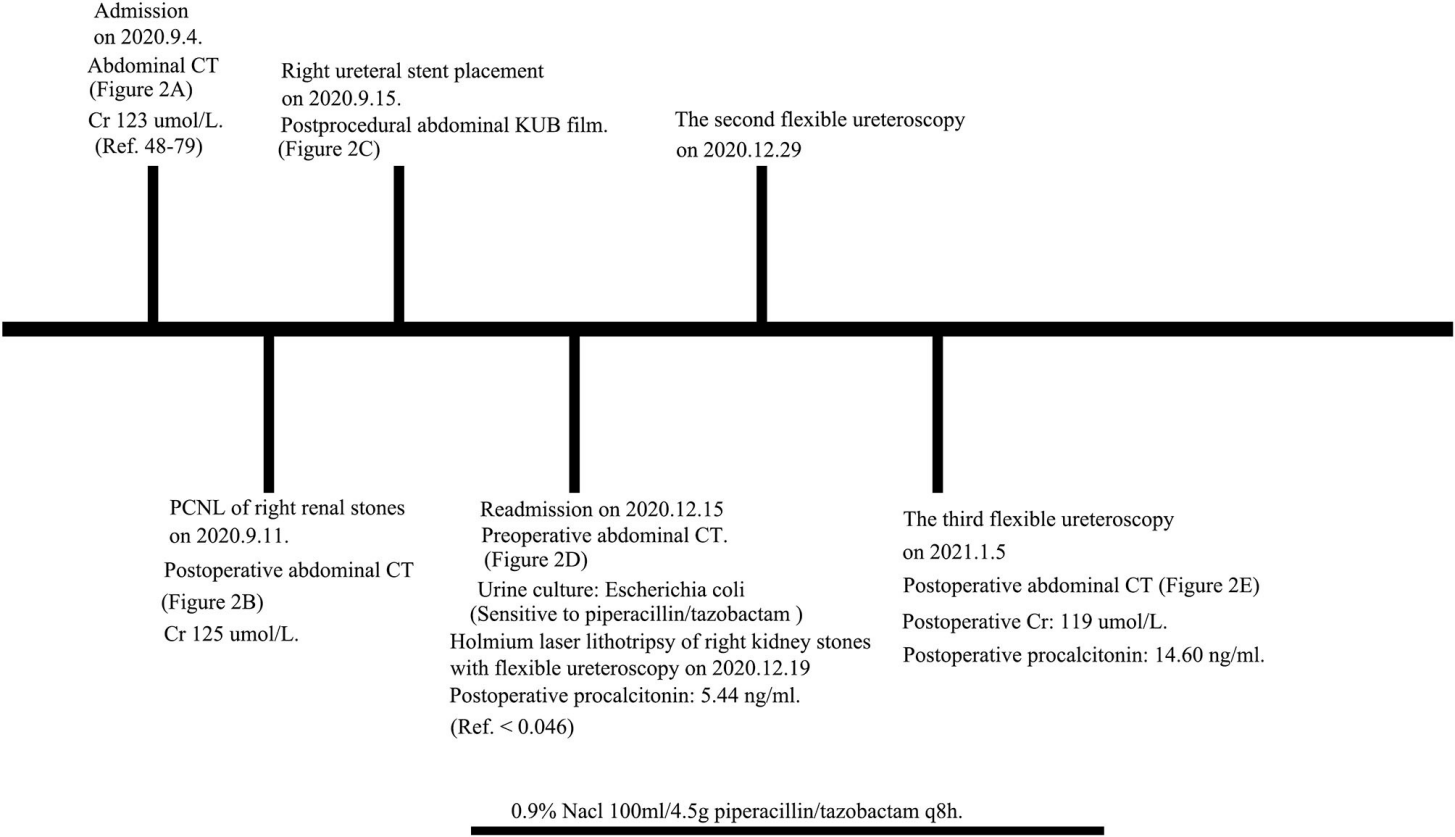
The timeline picture of this case. Cr, creatinine; PCNL, percutaneous nephrolithotomy; Ref., reference.

A 49-year-old female complaining of persistent pain in the right waist for more than 2 days came to our center. We detected no obvious abnormalities during the abdominal and skin examination. The patient underwent left kidney incision and stone removal in 2009, and the left kidney was already atrophy at the time of admission. Besides, the patient had a history of type 2 diabetes mellitus, but no obvious family history. The CT scans showed that the stone size was 4.51 × 4.17 cm. Before PCNL, cefoxitin was given for 7 days due to urinary tract infection and there was no evidence of pyonephrosis. PCNL of right renal stones was conducted after the relevant radiological examinations were completed. However, we had to terminate the operation due to severe bleeding during channel establishment. After 4 days, the patients underwent transurethral right ureteral stent placement under local anesthesia. The patient underwent holmium laser lithotripsy of right kidney stones with FURS 3-months later. We found that the stones were embedded with inflammatory matrix-like substances, which were cotton-like and quite soft. Desperately, holmium laser lithotripsy had very limited effect on the infectious staghorn stones. This patient underwent three laser lithotripsies with FURS. For the first time, we used the 14/16 Fr. vacuum-assisted sheath and 200 μm holmium laser fibers. An energy setting of 0.6 J and a rate of 60 Hz were applied (Lumenis Pulse 120H, Israel). The effect of the vacuum-assisted sheath was limited since the stones could not be effectively powdered. For the remaining two times, we used the 14/16 Fr. normal ureteroscope sheath and 200 μm holmium laser fibers (energy: 0.6 J; frequency: 60 Hz). During the procedure, 8.5 Fr digital FURS (Olympus®, Tokyo, Japan) and the Holmium Lumenis 120H® laser with 200-μm laser fibers from Lumenis® were used. The patient developed chills and fever during the three operations. We used piperacillin/tazobactam as an anti-infection treatment based on the results of the drug sensitivity test. Our postoperative CT scan revealed that most of the stones had been cleared, and the patient was discharged smoothly. The stone analysis indicated infectious stones which were consistent with the Hounsfield unit on CT. The CT images throughout the period are presented in Figure 1 and the timeline picture is shown in Figure 2. All the procedures performed in studies involving human participants were in accordance with the ethical standards of the institutional and/or national research committee(s) and with the Helsinki Declaration (as revised in 2013). A written informed consent was obtained from the patient.

## Discussion

Patients with solitary kidney are more susceptible to deterioration in renal function (1). In addition, they are more likely to have bleeding and blood transfusions than those with double-sided kidney stones, because the solitary kidneys have a greater percentage of blood flow around the renal collection system as a result of increased compensation (6). There is no doubt that uncontrollable renal bleeding is a life-threatening complication for patients with solitary kidney. Thus, the treatment of patients with solitary kidney having complex staghorn stones is one of the most challenging problem in urology. The PCNL procedure of the patient in this paper was suspended for the first time because of severe bleeding during the establishment of the puncture channel. We believed that the obvious compensatory hyperplasia of the right kidney caused this result. In this circumstance, we decided to use a flexible ureteroscope to treat the stones. Unexpectedly, renal stones of the patient were not only overburdened and infectious but also resistant to holmium laser due to floppy property.

We found the following techniques which could promote the expulsion of stone fragments from the sheath during the third procedure. First, we divided larger stones into several pieces and thereby extended the fiber to pull the smaller stone to the opening of ureteroscope sheath. Subsequently, we used the sheath to press the stone. It might be necessary to adjust the position of the sheath constantly. Second, we used a holmium laser with intermittent high frequency (60 Hz) to oscillate the stone, and the fallen pieces could be washed out along the sheath. We proposed that the vortex formed by the oscillation might contribute to the removal of stones. Besides, flexible ureteroscopy forceps also helped to pinch out soft stones. Of note, thick sheath is essential for this special situation, otherwise, open surgery might be an alternative. Through this case, we found that the combination of intermittently high-frequency oscillation and FURS forceps, helping to remove the stone fragments, might contribute to treat the complex infectious stone in a patient with solitary kidney. Additionally, the patient is also satisfied with the results of the operation.

We do have the following limitations. First, as solitary kidney with staghorn floppy stones is extremely rare and the clinical trials with a large sample size are difficult to conduct, we only reported a single case, providing limited evidence for the management of such case. However, our technique points might provide a reference when similar patients are encountered again in clinical practice. Second, we did not perform a double-J stent placement immediately after the PCNL, because we thought right renal artery superselective interventional embolization might be necessary and we believed that we did not enter the collection system. In retrospect, we should perform it at the same time during the failed PCNL, and mini-invasive PCNL might be worth a try. The use of retrograde intrarenal surgery in a patient with a stone >2 cm is associated with infectious complications, as in this case, due to the development of high intrarenal pressures and pyelovenous backflow with subsequent entrance of microorganisms into the bloodstream. Therefore, the use of three sessions of RIRS in patients with solitary kidney and staghorn stone disease should be cautiously used. The technique in this study should be used in specific situations, such as failure of PCNL. Besides, antibiotics were essential throughout the perioperative period. Third, basket stone retrieval device might replace grasper, and it is worth trying in clinical practice. Lastly, the infectious stones are more subject to relapse, and the key to management is how to prevent the recurrence of stones.

## Conclusions

We found that the combination of intermittently high-frequency oscillation and FURS forceps might contribute to treat the complex infectious stone in a patient with solitary kidney.

## Data Availability Statement

The raw data supporting the conclusions of this article will be made available by the authors, without undue reservation.

## Ethics Statement

The authors are accountable for all aspects of the work in ensuring that questions related to the accuracy or integrity of any part of the work are appropriately investigated and resolved. All procedures performed in studies involving human participants were in accordance with the ethical standards of the institutional and/or national research committee(s) and with the Helsinki Declaration (as revised in 2013). The patients/participants provided their written informed consent to participate in this study. Written informed consent was obtained from the patients/participants for the publication of any potentially identifiable images or data included in this article.

## Author Contributions

DF and WW contributed to conception and design of the study. DF organized the database and wrote the first draft of the manuscript. All authors contributed to manuscript revision, read, and approved the submitted version.

## Funding

This work was supported by the Department of Science and Technology of Sichuan Province, China (2020YFH0099).

## Conflict of Interest

The authors declare that the research was conducted in the absence of any commercial or financial relationships that could be construed as a potential conflict of interest.

## Publisher's Note

All claims expressed in this article are solely those of the authors and do not necessarily represent those of their affiliated organizations, or those of the publisher, the editors and the reviewers. Any product that may be evaluated in this article, or claim that may be made by its manufacturer, is not guaranteed or endorsed by the publisher.
